# Unveiling the enhanced structural, elastic, mechanical, and optoelectronic properties of BaWO_4_*via* oxygen vacancies and europium doping: a DFT + *U* insight into tailored energy applications

**DOI:** 10.1039/d5ra01743b

**Published:** 2025-06-04

**Authors:** Shah Hussain, Raj Wali, Sikander Azam, Qaiser Rafiq, Mehmoona Nisar, Wilayat Khan, Yasir Saeed, Mohammed A. Amin

**Affiliations:** a Department of Physics, Abbottabad University of Science and Technology Abbottabad Pakistan yasir.saeed@kaust.edu.sa; b National Water and Energy Center, United Arab Emirates University Al Ain 15551 United Arab Emirates; c Department of Physics, Riphah International University Islamabad Pakistan sikander.physicst@gmail.com; d Department of Physics, Bacha Khan University Charsada Pakistan; e Department of Chemistry, College of Science, Taif University P.O. Box 11099 Taif 21944 Saudi Arabia

## Abstract

In this study, we examine the structural, electronic, and optical properties of Eu-doped BaWO_4_ using the full potential linearized augmented plane wave (FPLAPW) method, within the framework of density functional theory (DFT). The calculations are performed using the Generalized Gradient Approximation with an optimized effective Hubbard parameter ‘*U*’ (GGA + *U*), implemented in WIEN2K software. The introduction of oxygen vacancies and Eu doping significantly impacts the elastic properties of BaWO_4_, including its elastic constants, bulk modulus, shear modulus, and Poisson's ratio. These modifications result in a predictable reduction in stiffness and rigidity but enhance the material's optoelectronic functionality. By adding the Hubbard parameter term ‘*U*’, with a value of *U* = 7 eV, a more accurate description of the system is achieved, particularly in systems with a strong correlation of d- and f-electronic states. In contrast to the wide band gap (4.885 eV) of parent BaWO_4_, the electronic band gap decreases to 2.80 eV for Eu-doped BaWO_4_. Additionally, the creation of O-deficiency in BaWO_4_ results in a reduction in the band gap value to 0.8 eV (spin-up) and 2.6 eV (spin-down). The partial density of states (PDOS) reveals that the Eu-f (Eu-d) state dominates the valence band maximum, while the conduction band minimum is attributed to the W-d state for spin-up and spin-down channels, respectively. Further analysis of the optical response, including the dielectric constant *ε*(*ω*), absorption coefficient *I*(*ω*), reflectivity *R*(*ω*), refractive index *n*(*ω*), and optical energy loss functions *L*(*ω*), with different incident photon energies, is presented. When Eu atoms are added to the BaWO_4_ sample, the gap between optical bands narrows, indicating the development of intermediate energy levels. The calculated band gaps confirm that the *E*_g_ of oxygen vacancy (V_O) < oxygen vacancy shows good agreement with optoelectronic devices.

## Abstract 2

Eu doping and oxygen vacancies in BaWO_4_ reduce the band gap and enhance optoelectronic properties. DFT + *U* calculations show improved absorption and dielectric response, making it a promising material for energy-efficient optical devices.

## Graphical abstract

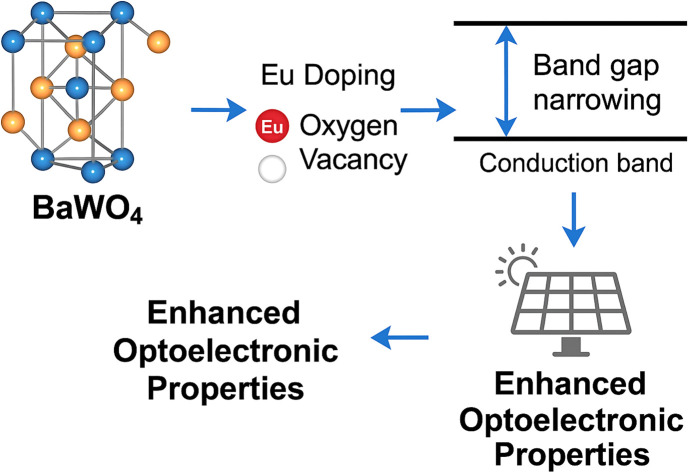

## 1 Introduction

Dye-sensitized solar cells (DSSCs) have gained popularity in recent decades as a green energy source due to their low cost, high stability, ease of processing, and high conversion efficiencies (PCEs), making them a part of the next generation of photovoltaic cells.^1–4^ DSSCs typically use a photoelectrode, a counter electrode, and a redox electrolyte comprising iodide and tri-iodide ions.^5,6^ The photoelectrode plays a crucial role in the generation of photo-excited electrons in DSSC performance. O'Regan and Gratzel published the first studies on DSSCs in 1991,^7^ based on a visible light-sensitizing material using TiO_2_ nanoparticles, with a conversion efficiency of 7%. Methods such as surface modification, surface treatment, semiconductor recombination, luminous materials, and structural optimization have been explored to improve photoelectrode efficiency.^8–12^ The amount of light energy absorbed by the DSSC is strongly influenced by the photoelectrode's light absorption spectrum.^13^

Recently, luminescent conversion materials have been proposed as a viable option for extending light absorption in DSSCs.^14^ These materials can shift or convert a broad spectrum of light into photons of a single wavelength. Up-conversion (UC), down-conversion (DC), and down-shifting (DS) luminescence techniques are used to transform infrared or ultraviolet light into visible light.^15^

Down-shifting is a single-photon process that involves converting one high-energy absorbed photon into a low-energy photon. The Stokes Law is demonstrated by the Stokes shift, which represents a wavelength change. One high-energy photon can be converted into one or more low-energy photons using down-converting materials. Both techniques help reduce the energy loss caused by the thermalization of hot charge carriers after high-energy photon absorption.^16,17^ Rare earth (RE) ions have been effectively employed in dye-sensitized solar cells to improve light scattering and absorption spectra of dye-loaded photoactive electrodes in DSSCs.^18^ As a result, down-converting materials have the potential to enhance the efficiency of DSSCs.^14,19^ Praseodymium (Pr^3+^)-doped inorganic host materials are among the most promising white-light-emitting nanophosphor materials because they can provide broad and intense luminescence in both visible and infrared regions, making them more suitable for a wider range of optical applications.^20^

Due to their applications in sensor technology, laser materials, color displays, optoelectronics, white light-emitting diodes, bio-imaging, and security applications, barium tungstate (BaWO_4_ or BWO) is the primary host material for rare earth ions.^21–24^ Rare earth ion-doped barium tungstate possesses exceptional thermal and chemical stability, along with distinctive electrical and optical properties resulting from different energy level configurations. BaWO_4_ has been widely used as a host material for various phosphor powders, including green and bluish phosphors. Barium tungstate (BaWO_4_) is the most well-known and heaviest member of the alkaline earth tungstate family. BaWO_4_ is one of the most reactive alkaline earth tungstates, widely investigated due to its excellent electrical conductivity and photoluminescence properties. These compounds play a significant role in various technological applications such as light-emitting diodes (LEDs), scintillator detectors, and solar cells. Eu- and Sm-doped BaWO_4_ is synthesized using the solid-state method and characterized by X-ray diffraction. Experimental findings, such as optical properties, luminescence intensity, photoluminescence (PL) lifetime, stimulated emission cross-section, bandwidth, and quantum efficiency of doped BaWO_4_, were improved with Eu and Sm doping.^25^ Cho^26^ experimentally studied the structural and optical properties of Eu-doped BaWO_4_, finding that Eu incorporation increased emission intensity. S. Cho^27^ developed low-cost BaWO_4_:RE^3+^ (RE = Eu or Sm) phosphor powders using a solid-state reaction with various doping concentrations of RE^3+^. They studied the surface morphology and photoluminescence spectra of the Eu^3+^-doped BaWO_4_. The photoluminescence spectra of Eu^3+^-doped BaWO_4_ occurred at 274 nm, while for Sm-doped BaWO_4_, it occurred at 568, 603, and 649 nm, respectively. Deng *et al.*^28^ prepared a quasi-single crystalline thin film, which is a promising material for white-light-emitting diodes (w-LED) for solid-state lighting, displays, and photoelectric detectors. They discussed the mechanism of energy transition and luminescence kinetics of the doped BaWO_4_ matrix. George *et al.*^29^ presented a new perspective on the highly stable rare-earth-doped BaWO_4_ nanofibers, emphasizing the reversibility of fluorescence quenching, which allows precise reuse during the sensitive detection of explosives, compared to other materials. Although much experimental work has been done, little theoretical work has been conducted to explore the physical properties of pure BaWO_4_. Carvalho *et al.*^30^ computed the phonon density of states, specific heat at constant volume, entropy, and Gibbs free energy of the three MWO_4_ (M = Ca, Sr, or Ba) compounds. It was found that the magnitude of the specific heat at constant volume at high temperature follows the order: BaWO_4_ > CaWO_4_ > SrWO_4_. Additionally, a relationship between the M^2+^ ionic radius in the scheelite MWO_4_ (M = Ba, Sr, or Ca) and their entropy, Gibbs free energy, and Helmholtz free energy was verified. Ashraf *et al.*^31^ investigated the electronic and optical properties of BaWO_4_ using Density Functional Theory (DFT) within the framework of CASTEP at an optimized cut-off energy value of 1200 eV. They found that the highest band gap value of BaWO_4_ corresponds to the highest loss function and the smallest value of the dielectric constant. High absorption in the UV range and a large refractive index make BaWO_4_ suitable for the formation of sensors, filters, transparent conducting films for window layers on solar cells, solar control coatings, and scintillators. Despite many experimental and theoretical works, the role of O-deficiency on the optoelectronic behavior of Eu-doped BaWO_4_ using the Generalized Gradient Approximation (GGA) + Optimized Effective Hubbard Parameter (*U*) (GGA + *U*) approach remains unexplored. This research investigates the structural, electronic, and optical properties of the compound Ba_1−*x*_Eu_*x*_WO_4_ (*x* = 2.08%) with oxygen vacancy (V_O). Here, Generalized Gradient Approximation (GGA) + Optimized Effective Hubbard Parameter (*U*) (GGA + *U*) is employed to account for d-state or f-state corrections to the electronic structure. Adding the Hubbard potential term “*U*” further enhances the physical properties, in addition to the electronic structure, including structural and optical properties. The objective of this work is to provide a comprehensive description and correction of the structure, electronic, and optical properties of Eu-doped BaWO_4_ and O-defect when the Generalized Gradient Approximation (GGA) + Optimized Effective Hubbard Parameter (*U*) (GGA + *U*) approximation is employed in WIEN2K software. This study will help establish a roadmap for the development of new optoelectronic devices and light-emitting diodes (LEDs). By using Generalized Gradient Approximation (GGA) + Optimized Effective Hubbard Parameter (*U*) (GGA + *U*) in WIEN2K software, we compare the results of electronic properties, including the partial density of states (PDOS), the total density of states (TDOS), and optical properties of Eu-doped BaWO_4_ and O-defects with those of pure BaWO_4_.

## 2 Computational methodology and structural details

### 2.1 Structural properties

**Fig. 1:**
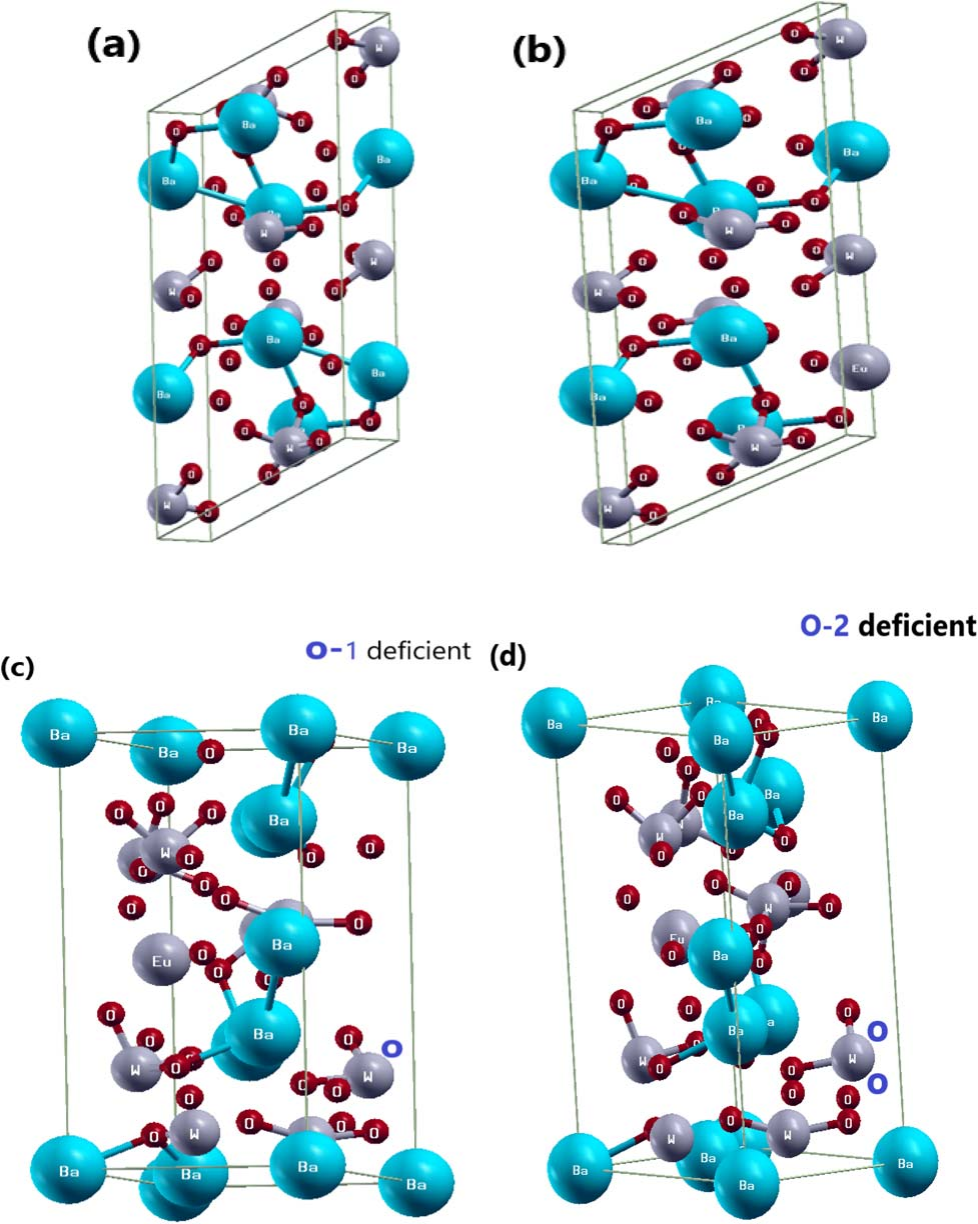
Optimized unit cell structure, (a) parental structure of BaWO_4_, (b) Eu-doped with Ba_1−*x*_Eu_*x*_WO_4_ compound, (c) & (d) 1-oxygen atom deficient (V_O) & 2-oxygen atoms deficient (0–2) in Ba_1−*x*_Eu_*x*_WO_4_ compound.

In this work, we investigate the three-dimensional tetragonal structure of BaWO_4_ with the space group *I*4_1_/*a* (88) and crystallographic parameters *a* = 15.11, *b* = 14.43, *c* = 29.699, and *α*, *β*, *γ*. We also study doped BaWO_4_ with Eu, along with oxygen vacancy (V_O), which adopts a monoclinic layered crystal structure with the crystallographic parameters *a* = 14.43, *b* = 15.11, *c* = 26.72, and *α*, *β*, *γ*. The pure crystal structure of BaWO_4_ is shown in Fig. 1(a). Fig. 1(b) illustrates the doping of Eu (*x* = 2.08%) in the investigated compound Ba_1−*x*_Eu_*x*_WO_4_. In this research, we also introduce oxygen vacancy (V_O & 2V_O), and as the oxygen vacancy increases, the band gap of Ba_1−*x*_Eu_*x*_WO_4_ also increases. Fig. 1(c and d) shows the oxygen vacancy (V_O & 2V_O) in the Ba_1−*x*_Eu_*x*_WO_4_ compound. It is well-recognized that while the GGA + *U* method helps in correcting the self-interaction errors associated with localized d and f states, it may still underestimate the bandgap. More recently, self-interaction-corrected (SIC) methods have been developed, which offer a significant improvement in the prediction of electronic properties at a lower computational cost compared to hybrid functionals. These methods have demonstrated better agreement with experimental bandgaps in complex systems, as reported in previous studies.^32,33^ However, the main focus of the present study is not on achieving the most accurate bandgap values but rather on understanding the trends in structural, electronic, and optical properties upon Eu doping in BaWO_4_. All theoretical calculations of Eu-doped BaWO_4_ (*x* = 2.08%) and oxygen vacancies (V_O & 2V_O) were carried out under the framework of Density Functional Theory (DFT). To study the electronic and optical properties of the Ba_1−*x*_Eu_*x*_WO_4_ compound, we used the all-electron full potential linearized augmented plane wave (FP-LAPW) method as implemented in the WIEN2K simulation package software.^34^ To address the issue of underestimating the band gap of materials, including 3d, 4d, 5d, and 4f electrons within DFT, we employed the generalized gradient approximation + optimized effective Hubbard parameter (*U*) (GGA + *U*)^35^ to account for the d- and f-transition states. Here, we doped Eu (*x* = 2.08%) in BaWO_4_ and introduced oxygen deficiencies in Ba_1−*x*_Eu_*x*_WO_4_, reporting a brief description of its photoluminescence and electronic properties using Generalized Gradient Approximation (GGA) + Optimized Effective Hubbard Parameter (*U*) (GGA + *U*) within the framework of DFT. We also investigate the band gap of the Ba_1−*x*_Eu_*x*_WO_4_ compound and gain deeper insight into its structural and optoelectronic properties. In this study, we focus on the three-dimensional structures of BaWO_4_, Ba_1−*x*_Eu_*x*_WO_4_, and oxygen deficiency.

### 2.2 Optimized energy and volume

The structural properties of BaWO_4_ are obtained through the volume optimization technique. The optimization procedure in WIEN2K is highly accurate, allowing the calculation of the structural properties of materials. Structural optimization, based on Murnaghan's equation, was performed to obtain a relaxed structure. In this procedure, the optimized energy is extracted from a particular optimized volume, and these data are fitted using Murnaghan's equation of state to derive structural parameters such as the minimum total energy (*E*_o_), equilibrium volume (*V*_o_), bulk modulus (*B*, GPa), and *B*′.

**Fig. 2:**
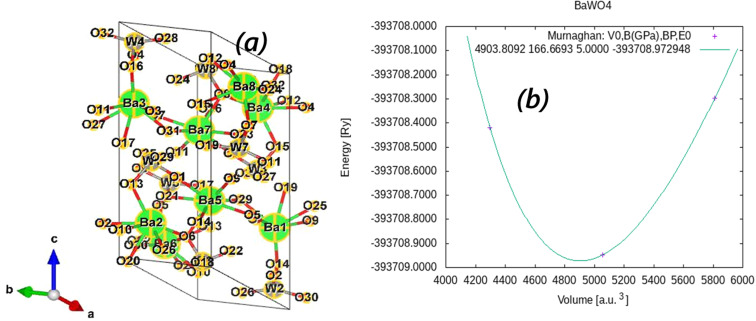
(a) Schematic of the single unit cell structure of BaWO_4_ (b) variation of total energies by volume for BaWO_4_ compound.

The crystal structure and optimization curve of BaWO_4_, plotting energy (RY) *versus* volume (Å^3^), are shown in Fig. 2(b). In this figure, it can be seen that the maximum energy is −5 348 328.00 eV at a volume of 4000 Å^3^. This *E*–*V* curve indicates that as the volume increases, the energy decreases, and when the volume approaches 4900 Å^3^, a maximum decrease in energy is observed at −5 348 341.92 eV. At higher values of volume, a rapid increase in energy is observed. The optimized ground-state properties, such as *V*_o_, *B* (GPa), *B*′, and the minimum energy (*E*_o_), are shown in Table 1. Additionally, the charge neutrality in Eu-doped BaWO_4_ is achieved by the creation of O-vacancies. The volume optimization of Eu doping, along with oxygen vacancies (V_O & 2V_O), at different positions in BaWO_4_ was performed. The minimized energy of the most relaxed crystal structures of Eu doping and oxygen vacancies (V_O & 2V_O) in the lattice are shown in Fig. 1(b–d).

**Table 1:** Volume (*V*_o_), Bulk modulus *B* (GPa), BP and minimum energy (*E*_o_)

Current work	Volume (*V*_o_)	Bulk modulus *B* (GPa)	BP	Minimum energy (*E*_o_)
Generalized gradient approximation (GGA) + optimized effective Hubbard parameter (*U*) (GGA + *U*)	4904.1699	165.8149	5.0000	−5 348 341.72 eV

Furthermore, the formation energy for the dynamic stability of Ba_1−*x*_Eu_*x*_WO_4_ with Eu doping and oxygen vacancy (V_O) is calculated using the following equations.^36^

Where, E_*x*_Eu_*x*_WO_4_ with Eu doping and oxygen vacancy (V_O) denotes the total energy of Ba_1−*x*_Eu_*x*_WO_4_ with Eu doping and oxygen vacancy (V_O). E_*x*_Ba, E_*x*_Eu, E_*x*_W, E_*x*_O, and *n*_*x*_Ba, *n*_*x*_Eu, *n*_*x*_W, and *n*_*x*_O denote the energy and number of atoms of Ba, Eu, W, and O, respectively. The negative sign of formation energy confirms their stability; higher negativity indicates greater stability. Consequently, Oxygen vacancy (V_O) is more stable than Ba_1−*x*_Eu_*x*_WO_4_.

### 2.3 Elastic properties of BaWO_4_ under the influence of oxygen vacancies and Eu doping

Elastic properties are essential in understanding the structural integrity and response of materials to external stress. These properties determine how materials behave under various loading conditions, influencing their suitability for specific applications. Barium tungstate (BaWO_4_), with its tetragonal scheelite structure, is a material of interest due to its promising optoelectronic and mechanical characteristics. Modifications such as oxygen vacancies and doping with europium (Eu) can significantly influence these elastic properties, affecting both mechanical stability and functional performance.

The intrinsic elastic properties of a material are described by parameters like elastic constants (*C*_11_, *C*_12_, and *C*_44_), bulk modulus (*B*), shear modulus (*G*), and Young's modulus (*E*). These parameters are vital for applications where materials are exposed to mechanical stresses, such as in sensors, scintillators, and light-emitting devices. However, introducing oxygen vacancies and dopant atoms into BaWO_4_ can disrupt its lattice structure, leading to changes in bonding interactions and, consequently, in its elastic properties.

#### 2.3.1 Reduction in elastic constants (*C*_*ij*_)

The elastic constants, including *C*_11_, *C*_12_, and *C*_44_, provide insight into the stiffness of a crystal along different directions (values are given in Table 2). In pristine BaWO_4_, the high values of *C*_*ij*_ reflect strong bonding between the [BaO_8_] dodecahedra and [WO_4_] tetrahedra, contributing to its robust lattice structure. However, introducing oxygen vacancies creates structural distortions, disrupting the uniform lattice framework. This reduces the interatomic forces, leading to a decrease in the elastic constants. Similarly, Eu doping introduces local lattice strain due to the size mismatch between the Eu ion and the original Ba ion, further diminishing the *C*_*ij*_ values.

**Table 2:** Elastic properties and their variations

Property	Pristine BaWO_4_	BaWO_4_ with oxygen vacancies	Eu-doped BaWO_4_
*C*_11_ (GPa)	71	64	62
*C*_12_ (GPa)	52	49	42
*C*_44_ (GPa)	100	91	44
Bulk modulus (*B*, GPa)	98	87	82
Young's modulus (*E*, GPa)	144	131	125
Poisson's ratio (*ν*)	0.27	0.29	0.32
Hardness (*H*, GPa)	5.0	4.5	4.0

#### 2.3.2 Bulk modulus (*B*)

The bulk modulus, which measures a material's resistance to uniform compression, is directly influenced by the rigidity of the crystal lattice. Pristine BaWO_4_ exhibits a relatively high bulk modulus due to its dense and ordered structure. Oxygen vacancies reduce the material's density and disrupt cohesive energy, thereby lowering its bulk modulus. Eu doping exacerbates this effect by introducing additional strain and asymmetry within the lattice. Despite this reduction, the material retains sufficient compressive strength for use in non-load-bearing optoelectronic applications.

#### 2.3.3 Shear modulus (*G*) and Young's modulus (*E*)

The shear modulus reflects the material's ability to resist shearing forces, while Young's modulus measures its tensile stiffness. Both properties decrease in BaWO_4_ upon introducing oxygen vacancies and Eu doping. The reduction in G and E arises from weakened bonding interactions and increased lattice defects, which reduce the material's resistance to shape and volume changes under applied stress. Notably, the decrease is more pronounced in Eu-doped BaWO_4_ due to the higher degree of lattice distortion introduced by the larger dopant ion.

#### 2.3.4 Poisson's ratio (*ν*)

The Poisson's ratio provides information about the elastic deformability of a material, representing the ratio of lateral strain to axial strain under stress. In BaWO_4_, the introduction of oxygen vacancies and Eu doping results in a slight increase in *ν*, indicating that the material becomes more deformable. This is typical in systems where structural modifications reduce overall rigidity, making the material more anisotropic and elastic.

#### 2.3.5 Implications for applications

Despite the observed reductions in elastic properties, BaWO_4_ remains a suitable candidate for applications where mechanical performance is secondary to functional properties. For instance, the improved luminescence due to Eu doping and the electronic transitions facilitated by oxygen vacancies make BaWO_4_ valuable in optoelectronic devices. In these applications, the trade-off between structural rigidity and enhanced functionality is acceptable.

For future advancements, balancing mechanical and functional properties is essential. Strategies such as co-doping or advanced synthesis methods can mitigate the drawbacks associated with reduced elastic moduli while further enhancing the material's performance. Computational modeling and experimental validation can provide insights into optimal doping concentrations and defect configurations to achieve this balance. The elastic properties of BaWO_4_, including its elastic constants, bulk modulus, shear modulus, and Poisson's ratio, are significantly influenced by the introduction of oxygen vacancies and Eu doping. These modifications result in a predictable reduction in stiffness and rigidity but enhance the material's optoelectronic functionality. Understanding and optimizing these trade-offs are critical for tailoring BaWO_4_ for specific applications, particularly in the fields of photonics and energy storage.

### 2.4 Electronic band structures

The investigation explores Janus MnSeTe and MnSTe electronic and magnetic characteristics under biaxial strain conditions while also studying doping effects on spin polarization and magnetic anisotropy which results in spin gapless semiconductivity when strain is compressive.^37^ Bilayers of Janus FeClF exhibit crucial valleytronic application features that include both bipolar magnetic semiconducting properties and anomalous valley Hall effects.^38^ Strain and doping elements transform Janus FeClF into an optimal material for spintronic technologies because they boost the Curie temperature together with perpendicular magnetic anisotropy.^39^

To gain a better understanding of the relationship between band structures and optical properties of the investigated compound BaWO_4_ with Eu doping (*x* = 2.08%) and oxygen deficiency, Generalized Gradient Approximation (GGA) + Optimized Effective Hubbard Parameter (*U*) (GGA + *U*) has been used within the framework of DFT. The electronic band structure analysis suggests that the band gaps for indirect-band-gap semiconductors in Ba_1−*x*_Eu_*x*_WO_4_ with Eu doping (*x* = 2.08%) and oxygen vacancy (V_O) are slightly smaller than those determined from the absorption spectrum.^40^ Recent findings emphasize the potential of these indirect-band-gap semiconductors for photocatalytic applications due to their lower radiative recombination rate.^41^ Furthermore, observations of flat bands within the conduction band (CB) indicate the presence of a second phase, possibly explaining the emergence of superconductivity.^42–49^ This insight underscores the potential of Ba_1−*x*_Eu_*x*_WO_4_ with Eu doping (*x* = 2.08%) and oxygen vacancy (V_O) for advanced applications in ultra-powerful magnet technology. Despite the observable range of the bands, their broadening in the presence of oxygen vacancy (V_O) further enhances their suitability for advanced applications. Traditionally, solar cells utilize silicon (Si) and germanium (Ge) due to their abundance, self-passivation, and indirect band-gap nature. The operation of an indirect band-gap semiconductor in a solar cell involves both a photon and a phonon, inducing changes in momentum and energy during the transition from the valence band (VB) to the conduction band (CB). The inverse relationship between minority carrier diffusion length and absorber depth highlights the importance of having a sufficiently thick absorbing layer for efficient solar cells.^50^

Despite the lower light absorption characteristic of indirect semiconductors, their advantages, including longer recombination lifetimes and larger diffusion lengths, make them suitable for efficient solar cells. Indirect band-gap semiconductors find applications in thin-film solar cells due to their weaker light absorption, enabling a broader photon energy range, particularly accommodating lower-energy solar photons.^51–53^ Additionally, their defect tolerance and reduced heat generation make them crucial for durable solar cells, especially in concentrator photovoltaic systems. Tandem solar cells, incorporating materials with different band gaps, further optimize energy conversion across the solar spectrum, leveraging the benefits of indirect-band-gap materials.^54–56^

In the context of double perovskite halides, the term effective mass of charge carriers plays a crucial role in understanding the behavior of electrons or holes. The effective mass varies depending on the material and its composition, influenced by the band structure and the types of phosphors and elements present in LED materials. The calculation of the effective mass of charge carriers (electrons or holes) in Ba_1−*x*_Eu_*x*_WO_4_ with Eu doping (*x* = 2.08%) and oxygen vacancy (V_O) at high symmetry points of VBM and CBM involves the use of the *E*–*K* dispersion curve. The formula utilized for determining the effective mass is as follows:m* = (ℏ^2^/(d^2^*E*/d*K*^2^))where m* represents the effective mass of the charge carriers. The symbol ℏ denotes the reduced Planck's constant, which is defined as ℏ = *h*/2π, with *h* representing Planck's constant, *h* = 6.62607015 × 10^−34^ J s. The term (d^2^*E*/d*K*^2^) refers to the second derivative of the energy (*E*) with respect to the wavevector (*K*), which characterizes the curvature of the energy band. This curvature is crucial for determining the effective mass of the charge carriers, as it directly influences their response to external forces in the material's energy band structure. This formulation illustrates how the electronic properties of a material are influenced by the band structure and is essential for understanding charge carrier dynamics in solid-state physics. The computed effective mass values, presented in Table 2, reveal a reduced effective mass, beneficial for carrier transfer, making Ba_1−*x*_Eu_*x*_WO_4_ with Eu doping (*x* = 2.08%) and oxygen vacancy (V_O) highly promising for various solar applications. The ratio of the effective masses of holes to electrons (*m*_h_/*m*_e_) significantly impacts carrier mobility, influencing the relative mobility of electrons and holes in a semiconductor. A smaller *m*_h_/*m*_e_ ratio, indicative of reduced effective mass, generally results in higher carrier mobility, enhancing the overall performance of the semiconductor in solar applications.

**Fig. 3:**
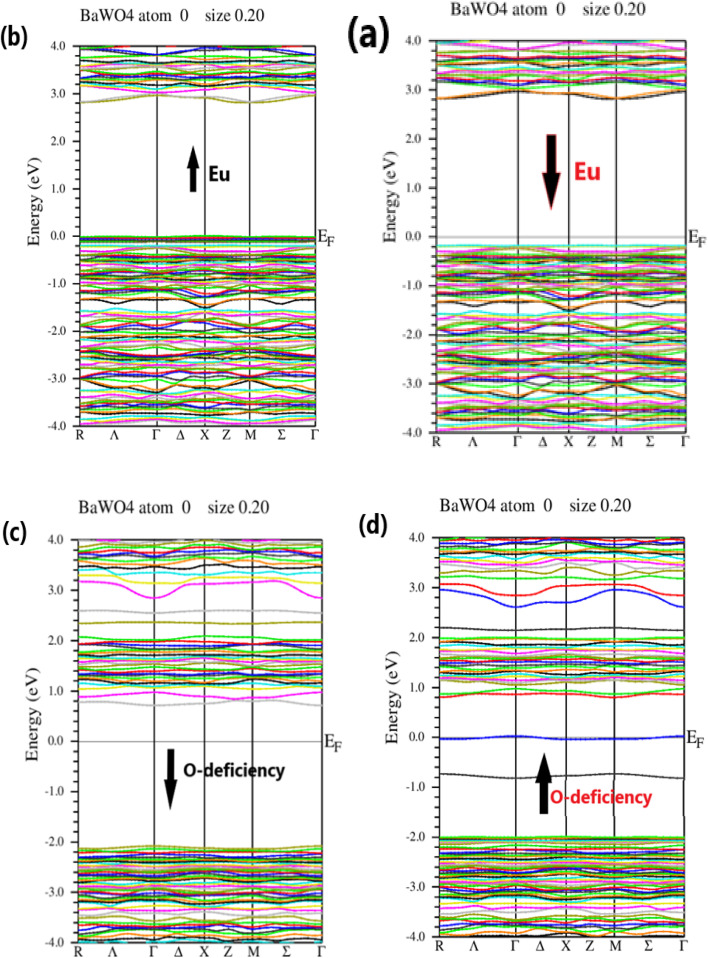
Energy Bandgap Structures, (a) & (b) represent the Ba_1−*x*_Eu_*x*_WO_4_ with Eu doped (*x* = 2.08%) for spin up state and spin down state, (c) & (d) represent the oxygen vacancy (V_O) in BaWO_4_ for spin up and spin down state along symmetry direction in Brillouin Zone.

The electronic structure of BaWO_4_ with Eu doping (2.08%) and oxygen vacancy along high symmetry direction points of the Brillouin Zone, such as *Γ*, *X*, and *M*, with energy plotted in electron volts on the *y*-axis for the spin-up and spin-down states, is depicted in Fig. 3 (a–d), respectively. The band gap of the parent compound BaWO_4_ is 4.885 eV. It is evident that in the Ba_1−*x*_Eu_*x*_WO_4_ compound with Eu doping (*x* = 2.08%), the spin-up and spin-down configuration graphs show that the maxima of the valence band and the minima of the conduction band do not lie on the same symmetric line, indicating that it is a semiconductor material with an indirect band gap, as shown in Fig. 3(a & b). The band gap energy is 2.8 eV for spin-up and 3.0 eV for spin-down states. From the band structures of the Eu-doped compound, we observe that the Fermi level shifts towards the valence band, which clearly indicates that the material is a p-type semiconductor, as depicted in Fig. 3(a & b).

Fig. 3(c & d) show the electronic band structure of the oxygen vacancy (V_O) in the BaWO_4_ compound for spin-up and spin-down states. This figure indicates that the maxima of the valence band and the minima of the conduction band lie on the same symmetric line, showing that it is a semiconductor material with a direct band gap. The band gap values for the spin-up and spin-down channels are 0.8 eV and 2.6 eV, respectively. It can also be observed that the Fermi level is slightly shifted towards the conduction band, indicating that the material is an n-type semiconductor for both spin-up and spin-down states. In the electronic band structure of oxygen deficiency, it is evident that there is a reduction in the band gap compared to the Eu-doped electronic band structure. The findings from the calculated band structures of the Eu-doped compound reveal not only a reduction in the band gap but also the formation of an intermediate band between the valence band (VB) and conduction band (CB) due to the doping of Eu into the host compound BaWO_4_. As the concentration of Eu atoms increases, the intermediate band also increases, and the fundamental band gap decreases. Consequently, this reduces the optical threshold, enhances the optical transition, and shifts it to the visible region, proving that Eu-doped BaWO_4_ is a promising material for optical devices.

### 2.5 Density of states

**Fig. 4:**
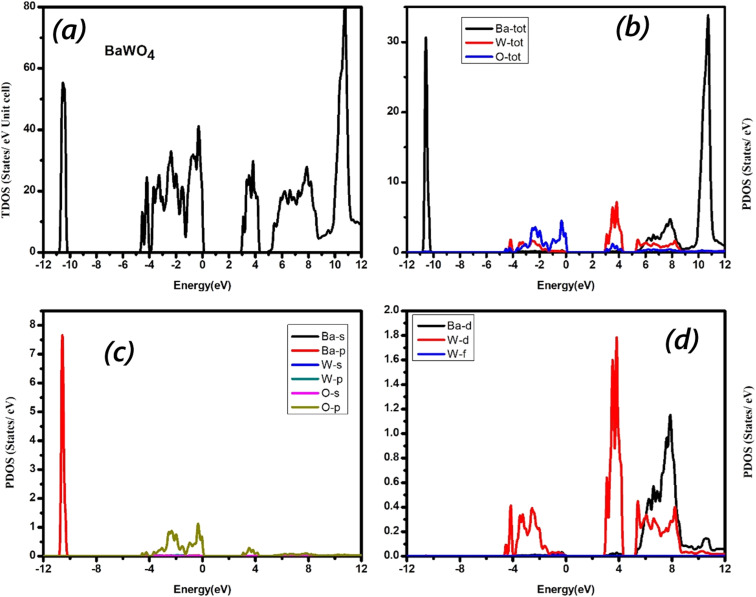
(a) Calculated total density of states, (b) the elemental resolved density of states, and (c & d) the orbital resolved density of states for BaWO_4_.

**Fig. 5:**
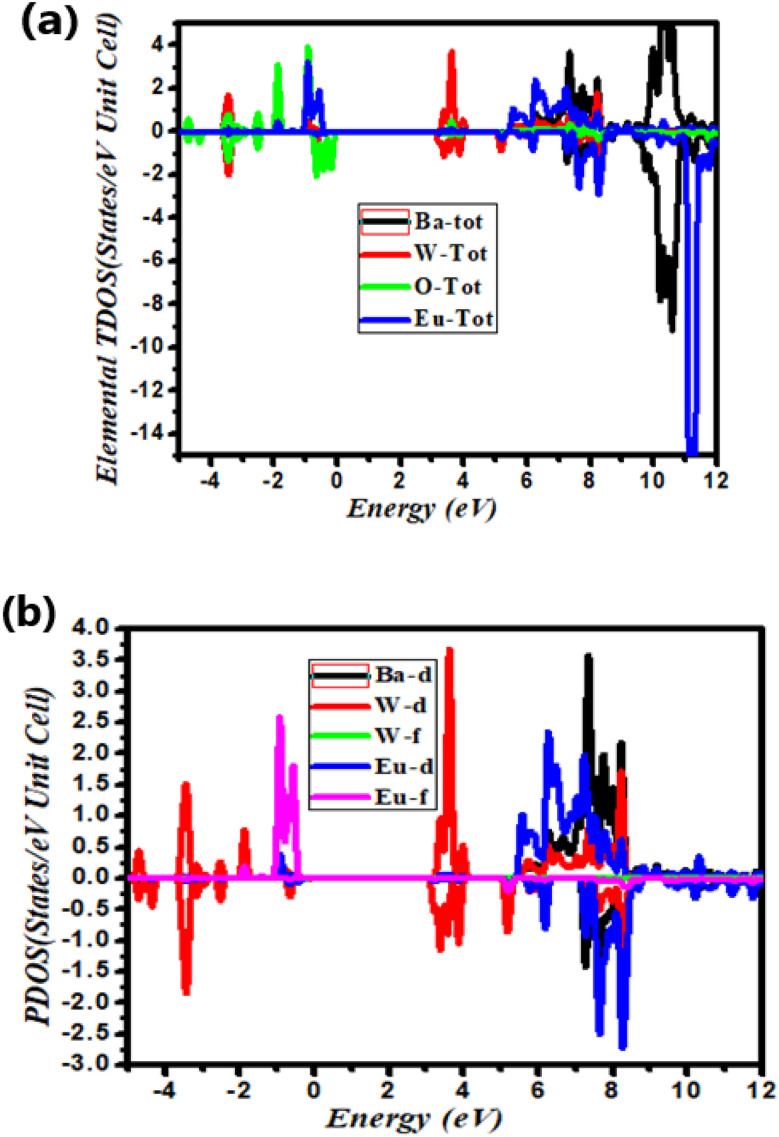
Density of states (states per eV per unit cell *vs.* energy (eV)) for the Ba_1−*x*_Eu_*x*_WO_4_ material with Eu doping (*x* = 2.08%). The figure includes (a) and (b) showing the Elemental Density of States (EDOS), elemental TDOS for Ba, W, O, and Eu, and the Partial Density of States (PDOS) for both spin-up and spin-down states.

Semiconducting materials demonstrate the typical electronic band structure, determined by the contribution of different electronic states in the conduction band and valence band. To study the changes in the electronic band structure, we calculated and plotted the total density of states (TDOS), elemental density of states (ETDOS), and partial density of states (PDOS) for the parent compound BaWO_4_, Ba_1−*x*_Eu_*x*_WO_4_ with Eu doping (*x* = 2.08%), and oxygen vacancy (V_O). The density of states (DOS) helps us understand the band energy nature and electronic parameters, such as the particular atomic and orbital origins of the material. To resolve the inconsistency between the minority and majority spin-up and spin-down states, the total density of states for the Ba_1−*x*_Eu_*x*_WO_4_ compound with Eu doping (*x* = 2.08%) has been computed using the Generalized Gradient Approximation (GGA) + Optimized Effective Hubbard Parameter (*U*) (GGA + *U*) approximation under the framework of Density Functional Theory (DFT). We also calculated the elemental total density of states (ETDOS) and partial density of states (PDOS) for the parent material BaWO_4_, Eu-doped (*x* = 2.08%), and oxygen-deficient materials depicted in Fig. 4 and 5. Partial density of states (PDOS) describes the contribution of different orbital states in both valence and conduction bands. The zero-energy level is known as the Fermi energy level. The region on the right side with positive energy values represents the conduction band, while the region on the left side with negative energy values represents the valence band. The graph below shows the spin-up and spin-down symmetries of the concerned material. In Fig. 4(a), the total density of states (TDOS) for the parent material BaWO_4_ reveals the maximum contribution near the Fermi level in the valence band, ranging from −5 eV to 0 eV. In the conduction band, the maximum contribution is observed in the region ranging from 10 eV to 12 eV. Fig. 4(b) represents the graphical representation of elemental total density of states (ETDOS) energy, ranging from −12 eV to 12 eV. It has been observed that in the valence band, the maximum contribution of oxygen near the Fermi level ranges from −4 eV to 0 eV, with a small contribution from tungstate (W) in the region from −5 eV to 0 eV. Also, a high contribution from barium (Ba) is observed in the region from −11 eV to −10 eV in the lower valence band. Fig. 4(c) is the graphical representation of orbital contributions from Ba (s-state & p-state), tungstate (s-state & p-state), and oxygen (s-state & p-states). Fig. 4(d) represents the graphical contributions of Ba (d-state) and tungstate (d-state & f-state) transition atoms in the valence and conduction bands of the compounds BaWO_4_ under investigation. Here, partial DOS was computed using the Generalized Gradient Approximation (GGA) + Optimized Effective Hubbard Parameter (*U*) (GGA + *U*) approximation for strongly correlated d-states and f-states of Ba and W in energy ranges from −12 eV to 12 eV. In Fig. 4(c), the valence band maximum contribution of the O-p state and the very small contribution of the O-s state can be seen near the Fermi level in the energy range from −4 eV to 0 eV, while the large contribution of the Ba-p state is observed in the energy range from −11 eV to −10 eV in the lower valence bands. In the conduction band, energy ranges from 0 eV to 4 eV, with a very small contribution from the O-p state. In Fig. 4(d), in the valence band, the major contribution of the W-d state is observed near the Fermi level in the energy range from −4 eV to 0 eV. In the conduction band, energy ranges from 3 eV to 4 eV with a maximum contribution from the W-d state, and the maximum contribution from Ba-d is seen in the energy range from 3 eV to 8 eV.

In Fig. 5(b), the elemental density of states (EDOS) for Ba_1−*x*_Eu_*x*_WO_4_ with Eu doping (*x* = 2.08%) for spin-up and spin-down states reveals that the maximum contribution near the Fermi level in the valence band occurs in the energy range from 0 eV to −1 eV. Furthermore, in the conduction band, the maximum contribution is observed in the energy range from 5 eV to 9 eV. Fig. 5(b) represents the elemental density of states (EDOS), ranging from −4 eV to 12 eV. It can be observed that in the valence band, the maximum contribution of oxygen for both spin-up and spin-down channels is near the Fermi level, while Eu contributes only to the spin-up channel and is completely absent in the spin-down channel. Tungstate (W) also contributes to the spin-up channel near the Fermi level. Here, it can be observed that the Eu total contribution is entirely absent in the spin-down state in the valence band, indicating that there is no mirror symmetry. Additionally, the elemental density of states (EDOS) in the conduction band shows a maximum contribution from W and a very small contribution from Eu and O near the Fermi level. In the energy range from 5 eV to 9 eV, Eu and W show strong hybridization for both spin-up and spin-down states.

Fig. 5(c) presents the graphical representation of the orbital contributions from Ba (d-state), tungstate (d-state & f-state), and Eu (d-state and f-states) transition atoms in the valence and conduction bands. Here, partial DOS was computed using the Generalized Gradient Approximation (GGA) + Optimized Effective Hubbard Parameter (*U*) (GGA + *U*) approximation for strongly correlated d-states and f-states of Ba, W, and Eu for both spin-up and spin-down states in energy ranges from −4 eV to 12 eV. In the valence band, the maximum contribution of the Eu-f state and a very small contribution of the Eu-d state for the spin-up channel can be seen near the Fermi level in the energy region from −1 eV to 0 eV, while a large contribution from the W-d state is observed in the energy range from −4 eV to −3 eV for both spin-up and spin-down channels. In the conduction band, the major contribution from the W-d state for both spin-up and spin-down states is observed near the Fermi level. The Eu-d state is slightly present in both channels of the conduction band. In the energy range from −0.6 eV to 8.5 eV, the Ba-d state is the maximum contributor for the spin-up state, and the Eu-d orbital shows a major contribution for both channels. In this energy range, Ba-d, W-d, and Eu-d states show strong hybridization. The maximum peaks of Ba-d for the spin-up state and Eu-d for both the spin-up and spin-down states can be observed. It can be noted that there is no mirror symmetry, and some magnetic moment exists in both spin-up and spin-down states. The magnetic moment increases with the concentration of Eu doping (*i.e.*, Eu1: 7.00005, Eu2: 14.00010). On the other hand, the creation of oxygen vacancy also increases the magnetic moment: as shown in Table 3: for V_O deficiency, the magnetic moment is 9.00029, and for 2V_O deficiency, it is 11.00057.

**Table 3:** Measured magnetic parameters for the Ba_1−*x*_Eu_*x*_WO_4_ (*x* = 2.08 and 4.16%) oxygen vacancy (V_O and 2V_O) in BaWO_4_

	Eu = 2.08%	Eu = 4.16%	Oxygen deficiency (V_O)	Oxygen deficiency (2V_O)		Eu = 2.08%	Eu = 4.16%	Oxygen deficiency (V_O)	Oxygen deficiency (2V_O)
μ^INT^	0.10175	0.20387	1.24118	2.37775	μ^O27^	−0.00286	−0.00287	0.04135	−0.00432
μ^Ba1^	−0.00001		0.00814	0.01441	μ^O28^	0.00000	−0.00157	0.00108	−0.00125
μ^Eu1^		6.92971			μ^O29^	−0.00016	−0.00485	−0.00444	0.02033
μ^Ba2^	0.00000	0.00004	0.00636	0.01440	μ^O30^	−0.00469	−0.00485	−0.00150	0.01667
μ^Ba3^	0.00004	0.00004	0.00013	0.00017	μ^O31^	−0.00156	−0.00157	0.01953	0.00328
μ^Ba4^	−0.00000	0.00004	0.00708	0.00749	μ^O32^	−0.00000	−0.00017	−0.00346	0.00336
μ^Ba5^	−0.00000	0.00015	0.00060	0.00751	μ^O33^	−0.00358	−0.00381	0.00301	0.00223
μ^Ba6^	0.00015	0.00015	0.00389	0.00383	μ^O34^	−0.00024	−0.00381	0.00037	0.00198
μ^Ba7^	0.00004	0.00004	0.00004	0.00370	μ^O35^	−0.00016	−0.00017	0.00249	−0.00094
μ^W8^	0.00001	−0.00037	0.68261	0.69100	μ^O36^	0.00000	−0.00004	0.00002	0.00025
μ^W9^	0.00002	−0.00038	0.00139	0.69155	μ^O37^	−0.00013	−0.00141	−0.00080	−0.01109
μ^W10^	−0.00039	−0.00038	0.01346	0.01265	μ^O38^	−0.00127	−0.00141	−0.00011	−0.01187
μ^W11^	−0.00038	−0.00037	−0.00024	0.01210	μ^O39^	−0.00004	−0.00004	0.00462	−0.00020
μ^W12^	0.00000	0.00031	0.04772	0.04751	μ^O40^	−0.00006	−0.00409	−0.00496	−0.00449
μ^W13^	0.00012	0.00014	0.00040	0.04720	μ^O41^	−0.00015	−0.00006		−0.00786
μ^W14^	0.00002	0.00014	0.00892	0.00796	μ^Eu41^			6.92979	
μ^W15^	0.00031	0.00031	0.00063	0.00832	μ^O42^	0.00009	−0.00006		−0.00774
μ^O16^	−0.00002	−0.00258	−0.00030	0.00392	μ^Eu42^			9.00029	
μ^O17^	−0.00007	−0.00082	0.00380	0.00509	μ^O43^	−0.00402	−0.00409	−0.00195	0.00640
μ^O18^	−0.00075	−0.00082	−0.00249	0.00489	μ^O44^	−0.00012	−0.00521	0.00003	0.00104
μ^O19^	−0.00254	−0.00258	0.00499	0.00005	μ^O45^	−0.00018	−0.00017	−0.00020	
μ^O20^	0.00000	−0.00351	−0.00016	−0.00341	μ^O46^	0.00001	−0.00017	−0.00400	
μ^O21^	−0.00009	−0.00008	−0.00006	0.00209	μ^O47^	−0.00510	−0.00521	−0.00604	
μ^O22^	0.00002	−0.00008	−0.00339	0.00166	μ^O48^			0.00006	
μ^O23^	−0.00350	−0.00351	0.00217	0.00143	μ^Eu48^	6.92984	6.92971		
μ^O24^	0.00000	−0.00287	−0.00035	−0.00134	μ^TOT^	7.00005	14.00010	9.00029	11.00057
μ^O25^	−0.00030	−0.00030	0.00168	0.05032					
μ^O26^	0.00000	−0.00030	−0.00283	0.05011					

One can observe that after Eu doping, the properties of the material are altered. In both spin-up and spin-down states, the valence and conduction regions are displaced from the Fermi level, indicating that the material behaves as a semiconductor.

## 3 Optical properties

The study of optical properties is essential for materials to be used in optoelectronic devices such as LEDs, solar cells, and other applications. To analyze the behavior of compounds for optical applications, a number of in-depth and comprehensive analyses have been conducted to determine their optical properties. These optical properties include dielectric function dispersion *ε*(*ω*), reflectivity *R*(*ω*), refractive index *n*(*ω*), absorption coefficient *I*(*ω*), and optical energy loss *L*(*ω*).

### 3.1 Dielectric function dispersion

Optical properties play a crucial role in understanding the nature of the material Ba_1−*x*_Eu_*x*_WO_4_ with Eu doping (*x* = 2.08%) and oxygen vacancies (V_O). The optical properties of a material represent the frequency response of its optical parameters, such as energy band structures, lattice vibrations, impurity levels, and magnetic excitations. Energy band structures and the dielectric dispersion function are interdependent parameters. Both interband and intraband transitions are important for defining the optical response of the material. Generally, intraband transitions are related to metals, while interband transitions, such as direct and indirect transitions, are associated with semiconductor materials. These transitions are highly sensitive to electromagnetic (EM) interactions. In our calculations, scattering phonons contribute to interband transitions (indirect), which are responsible for the emission of electromagnetic interactions. The dielectric constant *ε*(*ω*), reflectivity *R*(*ω*), refractive index *n*(*ω*), absorption coefficient *I*(*ω*), and optical energy loss *L*(*ω*) are commonly used to analyze the optical response. The complex optical susceptibility dispersion *ε*(*ω*) consists of two parts: the real part (Re(ω)) and the imaginary part (Im(ω)), which describe the photon interaction (*E* = *hv*) with electrons and show the material's response.

The absorption coefficient *I*(*ω*) and refractive index *n*(*ω*) are associated with dispersed dielectric functions, *ε*_1_(*ω*) and *ε*_2_(*ω*) through Kramer kronig relation *i.e.*
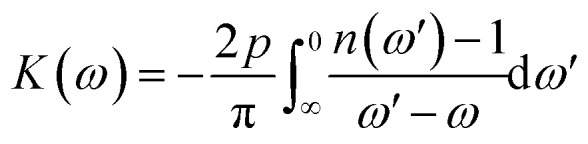
where *p* is the principle value of integral. The equation describes the frequency-dependent response function *K*(*ω*) of a system at a specific frequency *ω*. The term 2*p*/π is a normalization constant that simplifies to 2, ensuring proper scaling and dimensional consistency of the equation. The function *n*(*ω*′) represents a frequency-dependent property, such as the refractive index or dielectric function, evaluated at the integration variable *ω*′. This variable *ω*′ represents all possible frequencies that interact with the fixed frequency *ω*, and the integral sums the contributions from each of these frequencies. The fixed frequency *ω* is the point at which the system's response *K*(*ω*) is measured. The differential element d*ω*′ represents an infinitesimally small change in the frequency *ω*′, allowing the equation to capture the full range of contributions across all frequencies. This equation is crucial in understanding how the system interacts with different frequency components, often used in contexts like the Kramers–Kronig relations or in the study of optical properties and material responses. The complex optical function dispersion *ε*(*ω*) can be written as,*ε*(*ω*) = *ε*_1_(*ω*) + *iε*_2_(*ω*)

Dispersed dielectric function *ε*(*ω*) includes both real *ε*_1_(*ω*) and imaginary parts *ε*_2_(*ω*).*ε*_1_(*ω*) = *n*^2^ − *k*^2^

Equation describes the real part of the permittivity, where *ε*_1_(*ω*) represents the material's response to an electric field at frequency *ω*. Here, *n* is the refractive index, indicating the material's light bending ability, and *k* is the extinction coefficient, which measures how much light is absorbed by the material. This equation links the material's optical properties, such as refraction and absorption, to its overall electromagnetic response. The real part (Re(*ω*)) explains the dispersion and polarization when impinging light falls on these materials.

While the imaginary part (Im(*ω*)) represents the absorption of light, energy loss, and the sum of all transitions between occupied and unoccupied states. It also involves the calculations of the electronic wave function and energy eigenvalues.*ε*_2_(*ω*) = 2*nk*

Equation represents the imaginary part of the complex permittivity, where *ε*_2_(*ω*) describes the material's absorption response at frequency *ω*. In this equation, *n* is the refractive index, indicating how much light is refracted in the material, and *k* is the extinction coefficient, which quantifies the material's absorption of light. This equation links the material's absorption characteristics to both its refractive index and extinction coefficient, crucial for understanding energy dissipation in optical materials.

### 3.2 Absorption coefficient *I*(*ω*)

**Fig. 6:**
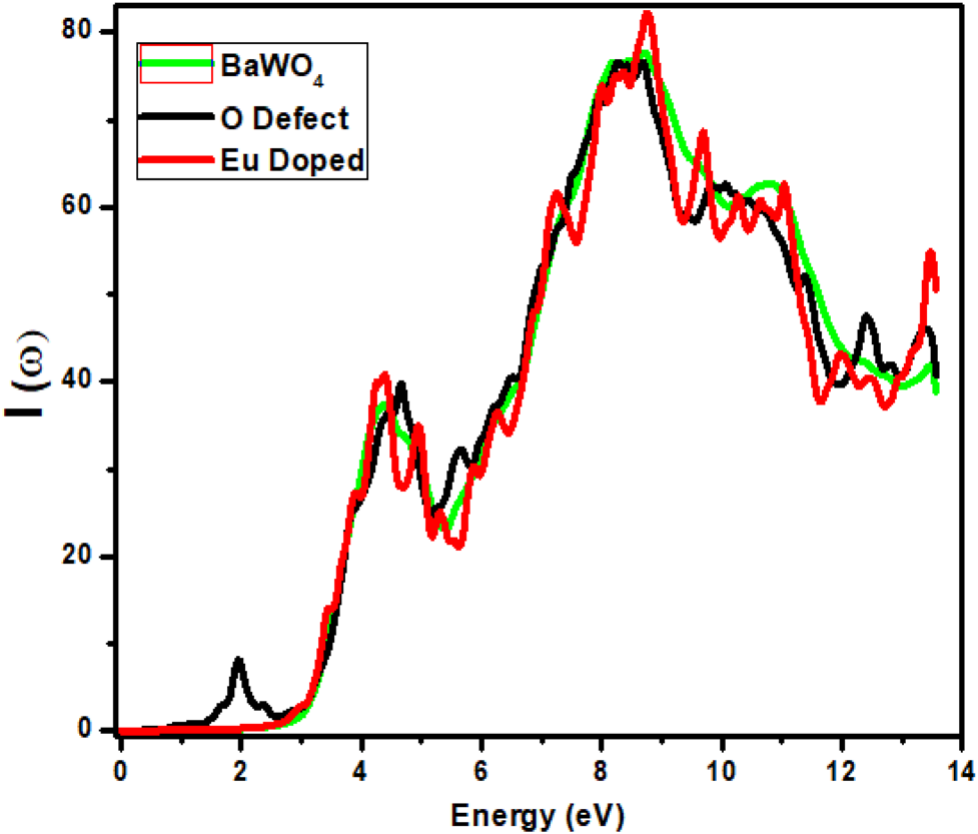
Calculated absorption coefficient *I*(*ω*) of Ba_1−*x*_Eu_*x*_WO_4_ with Eu-doped (*x* = 2.08%) and oxygen vacancy (V_O).

The absorption coefficient explains the penetration of light into the material before it is absorbed (or fully immersed into the material). Electronic transport cannot occur from the valence energy band to the conduction energy band below the band gap of the incident energy. Fig. 6 depicts the relationship between the absorption coefficient and energy (eV) for the parent structure BaWO_4_, oxygen-deficient, and Ba_1−*x*_Eu_*x*_WO_4_ compounds with Eu doping (*x* = 2.08%).

For BaWO_4_ and Ba_1−*x*_Eu_*x*_WO_4_ compounds, the static graph is observed from 0–2.8 eV. We can see that no absorption occurs in the infrared (IR) region. Absorption begins in the visible region for the parent compound BaWO_4_ and the doped compound Ba_1−*x*_Eu_*x*_WO_4_ after 2.8 eV, with the first peak observed at 4.5 eV, showing a small peak difference. For Eu-doped, the maximum peak value is observed at 9 eV in the ultraviolet region, where the absorption coefficient is 81%. For oxygen-deficient material, a static region (with no variation) is observed up to 1.5 eV, and the first peak appears in the visible region at 2 eV, where the absorption coefficient is 8%, which reduces the band gap and is beneficial for optoelectronic devices. For the parent material BaWO_4_ and the oxygen-deficient material, the maximum peak is observed at the same energy value 8.2 eV in the ultraviolet region, where the corresponding absorption coefficient is 75%, due to the forbidden band gap between the valence bands and conduction bands. All materials exhibit maximum absorption in the ultraviolet region. After the energy value of 8.2 eV, a decrease is observed in all graphs, with small fluctuations.

It can be seen that the band gap is reduced by 0.8 eV in the oxygen-deficient material, compared to the parent material BaWO_4_ and Eu-doped (*x* = 2.08%) Ba_1−*x*_Eu_*x*_WO_4_ compound (2.8 eV). The absorption coefficient *I*(*ω*) is formulated as:
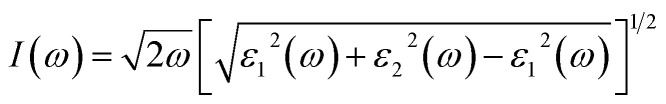
Equation describes the intensity *I*(*ω*) at a given angular frequency *ω*, accounting for both the real and imaginary components of the material's permittivity. The term *ε*_1_ represents the real part of the permittivity, related to the refractive index and how the material affects light propagation, while *ε*_2_(*ω*) is the imaginary part, linked to the material's absorption properties. The square root expression involving both *ε*_1_ and *ε*_2_ reflects the overall optical behavior of the material, combining refraction and absorption effects. This equation helps quantify how the material interacts with electromagnetic waves, providing insight into both transmission and absorption characteristics at a specific frequency.

### 3.3 Energy loss function *L*(*ω*)

**Fig. 7:**
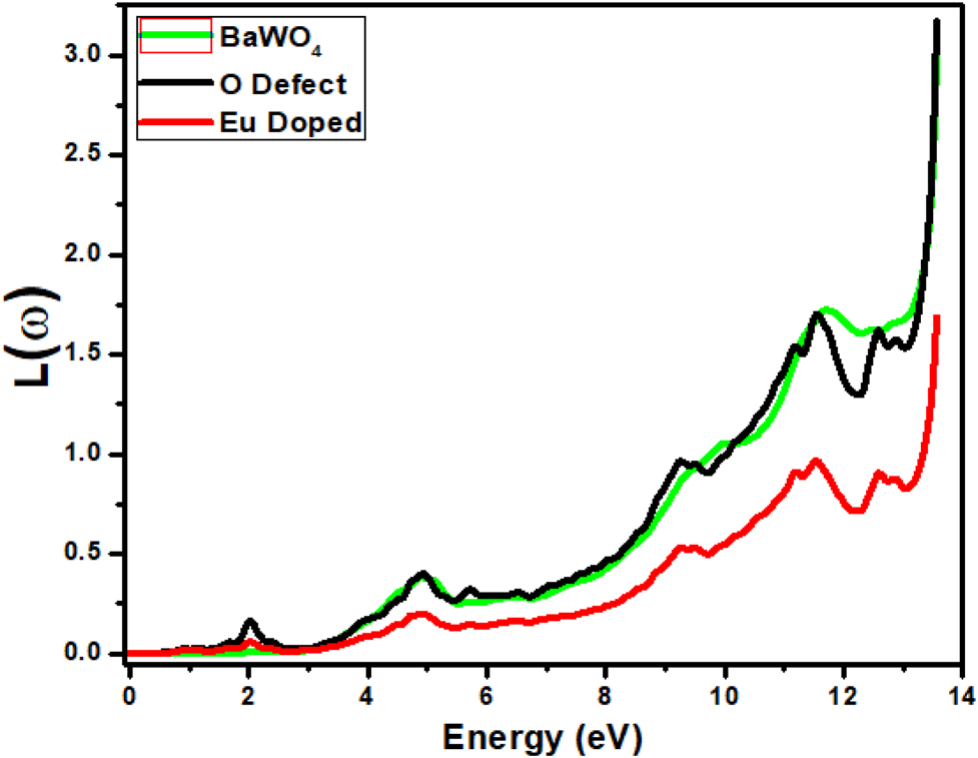
Calculated energy loss *L*(*ω*) of Ba_1−*x*_Eu_*x*_WO_4_ with Eu-doped (*x* = 2.08%) and oxygen vacancy (V_O).

The interaction of fast-moving electrons with material/matter results in energy loss of moving particles, given by the energy loss function *L*(*ω*). Fig. 7 shows the energy loss for the oxygen-deficient material shows the first peak at 2 eV in the visible region. The same material exhibits peaks at 4.8 eV, 9 eV, and 11.6 eV, and suddenly increases at an energy value of 13 eV. The major peaks for the parent material BaWO_4_ have been observed at 5 eV, and 11.8 eV, with a sudden increase at the energy value 13 eV. The major peaks observed for Eu-doped material are at 2 eV, 5 eV, 9.2 eV, and 11.9 eV, with a sudden increase at 13 eV. The overall graphs show that energy loss increases at higher energy values, and it is greater for the parent and oxygen-deficient materials as compared to the Eu-doped material. In the visible region, the maximum loss can be seen in the case of oxygen, where the maximum absorption is observed, indicating that oxygen is suitable for optoelectronic applications.

The relation of energy loss function *L*(*ω*) is
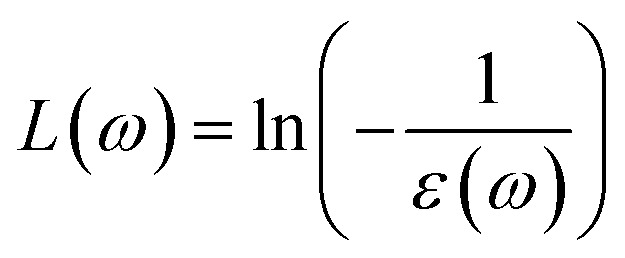
Equation describes the logarithmic function *L*(*ω*) of the permittivity *ε*(*ω*) at a specific frequency *ω*. In this context, *ε*(*ω*) represents the complex permittivity of the material, which accounts for both the real and imaginary components related to light propagation and absorption. The negative sign inside the logarithm indicates that the material exhibits some form of phase shift or inversion in its response at the frequency *ω*. This equation is useful for modeling the material's response in optical or electromagnetic systems, where the logarithmic relationship provides insights into the material's dispersive and absorptive properties.

### 3.4 Real and imaginary part dielectric function dispersion

**Fig. 8:**
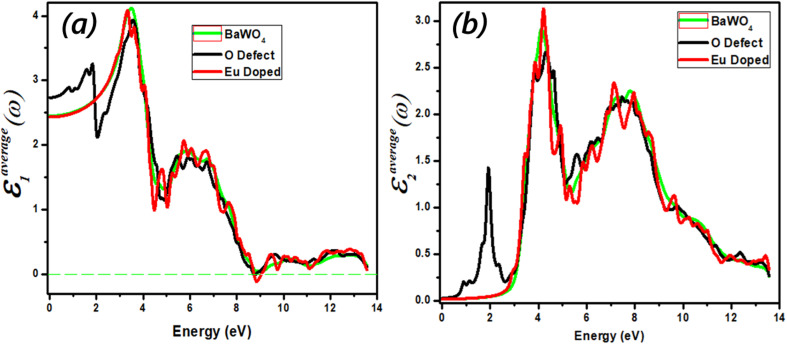
(a and b) Calculated optical spectra of Ba_1−*x*_Eu_*x*_WO_4_ with Eu-doped (*x* = 2.08%) and oxygen vacancy (V_O) (a) the real part *ε*_1_(*ω*) of the dielectric function, (b) the imaginary part *ε*_2_(*ω*) of the dielectric function.

The real part (Re(*ω*)) shows the refractive index of the material and describes the dispersion behavior of light. The real value (Re(*ω*)) of dispersed optical susceptibility is shown in Fig. 8(a). The dielectric function of the compound Ba_1−*x*_Eu_*x*_WO_4_ with Eu doping (*x* = 2.08%) and oxygen vacancy (V_O) is shown in Fig. 8(a) and (b). The real part of the dielectric constant (Re(*ω*)) first experiences a dramatic increase until it reaches its maximum value, after which it begins to decrease. The graph shows the band gap energy between 0–14 eV. The static values for BaWO_4_ and Eu-doped are 2.4, and for the O-defect are 2.7. As the static value of the O-defect is greater than the other two, it indicates that the O-defect is better suited for optoelectronic devices. The higher value of the dielectric constant of the O-defect shows that it has a smaller band gap, which further favors it over other materials for optoelectronic devices. The main peak is observed in the energy region from 2 eV to 4 eV. For Eu-doped, it is 3.1, for BaWO_4_ it is 3.2, and for the O-defect, it is 3.3, respectively. With a further increase in energy, the value of the real part (Re(*ω*)) decreases and enters the negative region at 8.9 eV, where the material shows metallic behavior.

The dielectric behavior of Ba_1−*x*_Eu_*x*_WO_4_ is slightly enhanced compared to oxygen vacancy (V_O). In the analysis of the compounds, there is a minimal variation in the energy loss function (*L*(*ω*)) within the range of −1.16 to −2 for energies greater than 10 eV. The negative values of (*L*(*ω*)) in this energy range indicate the metallic character of the compounds. Notably, the substitution of Ba_1−*x*_Eu_*x*_WO_4_ with oxygen vacancy (V_O) results in increased absorption peak intensities, shifting them to a lower energy region. This shift is attributed to the greater electronic influence of O compared to Ba_1−*x*_Eu_*x*_WO_4_ with Eu doping (*x* = 2.08%) and oxygen vacancy (V_O).

The relationship between static polarization and band gap follows Penn's model:^55^
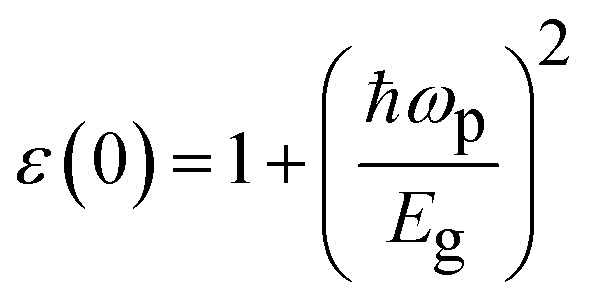
Equation describes the static dielectric constant *ε*(0) of a material at zero frequency. In this equation, ℏ represents the reduced Planck's constant, a fundamental constant in quantum mechanics. *ω*_p_ is the plasma frequency, which characterizes the natural oscillation frequency of the charge carriers (such as electrons) in the material. *E*_g_ is the band gap energy, representing the energy difference between the valence and conduction bands in semiconductors. The term 
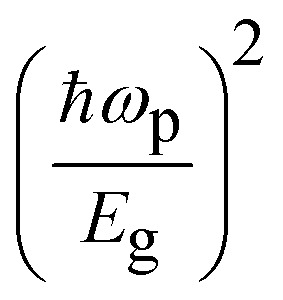
 quantifies the influence of the charge carrier's oscillations relative to the energy gap, affecting the material's dielectric response at low frequencies. This equation is particularly useful for modeling the optical properties of materials, especially semiconductors, under different conditions of frequency. As energy increases, the real part of the dielectric function becomes positive again, indicating the metallic nature of the material in the plasmon region. Plasmon oscillations arise from interband transitions and are associated with the dispersive part of the material in the optical spectra, causing electromagnetic waves to reflect back.

The imaginary part (Im(*ω*)) provides insights into the absorption coefficient of the material. It is crucial as it determines the maximum absorption area and governs interband transitions within the materials used for device fabrication. Due to the limitations of Density Functional Theory (DFT), the transition points (from the valence band to the conduction band) exhibit a slight deviation from the band structure and are illustrated in Fig. 8(b). The maximum value of (Im(*ω*)), known as the first absorption peak (FAP), corresponds to electronic transitions at the Fermi level. Fig. 8(b) represents the imaginary part of dispersed optical susceptibility (Im(*ω*)). Absorption starts in the IR region, and we observe a distinct main peak for oxygen-deficient material at 1.8 eV, with the corresponding dispersion value of 1.4, whereas there is no change in the behavior of other dopant materials. From 0 to 2.8 eV, no transition occurs for the Eu-doped material and the parent material BaWO_4_, due to the incident photon having less energy than the bandgap energy; in this region, no electron moves from the valence band to the conduction band. For the parent material BaWO_4_ and doped material, absorption starts after 2.8 eV in the visible region, and the maximum absorption is observed at 4 eV in the UV region. Maximum absorption is observed in the visible region for oxygen-deficient material.

### 3.5 Reflectivity *R*^avg^(*ω*) spectral

**Fig. 9:**
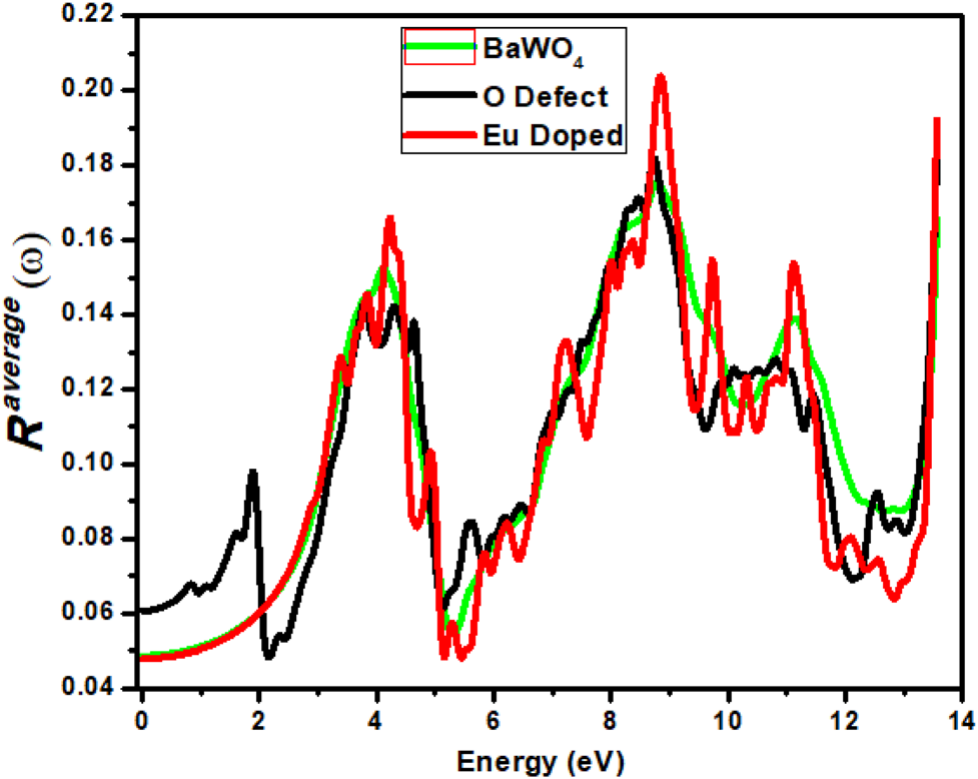
Calculated reflectivity *R*^avg^(*ω*) of Ba_1−*x*_Eu_*x*_WO_4_ with Eu-doped (*x* = 2.08%) and oxygen vacancy (V_O).

Reflectivity (*R*(*ω*)) *versus* photon energy gap (eV) is shown in Fig. 9. A constant value of reflectivity (*R*(*ω*)) has been observed in the energy range 0–1 eV in the infrared region for both the parent and Eu-doped materials, whereas for the oxygen-deficient material, it has been observed at 0.6 eV. The first peak for the oxygen-deficient material occurs in the visible region at 1.9 eV, known as the lossless region. For the parent material, the maximum reflectivity of 0.17 has been observed at an energy of 8.7 eV. For Eu-doped material, the maximum reflectivity of 0.20 is observed at an energy value of 8.85 eV, and for the oxygen-deficient (V_O) material, a high peak is observed at an energy value of 8.7 eV in the ultraviolet region, where the corresponding *R*(*ω*) is 0.18. A remarkable increase in reflectivity was observed for all materials after 5–9 eV and 13 eV, with small fluctuations associated with the indirect transition. A smaller value of reflectivity is observed in the visible region for the oxygen-deficient material at 2 eV, which suggests that the oxygen-deficient material is suitable for optoelectronics, due to the reduced bandgap of oxygen compared to Eu-doped materials. All materials show maximum reflectivity in the ultraviolet (UV) region; however, this reflectivity is insufficient for use in materials designed for shielding.

Reflectivity *R*^avg^(*ω*) is calculated by using the following eq.
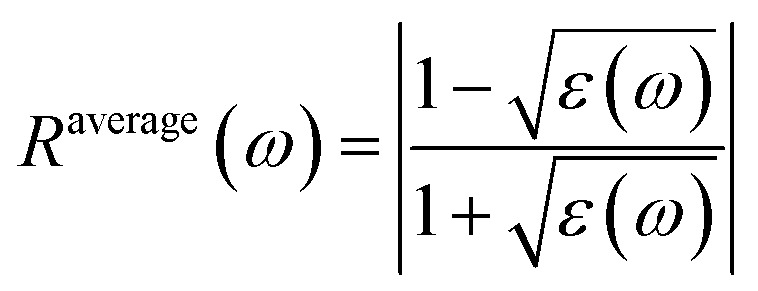
Equation describes the average reflection coefficient, *R*^average^(*ω*), of a material at a given frequency *ω*. It is a measure of how much of an incident wave is reflected by the material, which depends on its complex permittivity, *ε*(*ω*). The term *ε*(*ω*) modifies the reflection by accounting for both the material's refractive index and its absorption. The equation captures the material's impedance relative to free space, providing insight into the wave-material interaction, especially in optical and electromagnetic applications.

### 3.6 Refractive index *n*(*ω*)

**Fig. 10:**
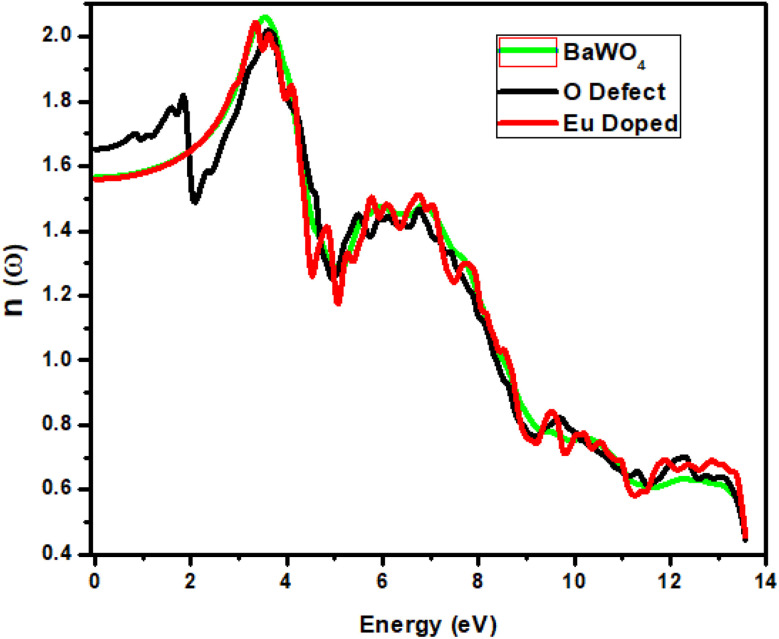
Calculated the refractive index of Ba_1−*x*_Eu_*x*_WO_4_ with Eu-doped (*x* = 2.08%) and oxygen vacancy (V_O).

The graph below shows the refractive index *n*(*ω*) in the energy range 0–14 eV. For the parent and Eu-doped materials, the static region lies between the energy ranges 0–1.7 eV in the infrared region. The static value of the refractive index *n*(*ω*) for oxygen-deficient material is 1.65, and for the parent compound BaWO_4_ and Eu-doped compound, it is 1.59. Fig. 10 shows the parent material graph reaches a maximum value of *n* = 2.1 at 3 eV. A decline in the graph for the parent and Eu-doped materials is observed at 5.2 eV. However, in the visible region, a sudden rise in the refractive index is observed from photon energies ∼2.1 eV, with an increase in the energy value. The maximum peak is observed at *n* = 2 at photon energy ∼3.5 eV in the ultraviolet region for both oxygen-deficient (V_O) and Eu-doped materials. A sudden decline in the refractive index is observed until it reaches 5.2 eV for both oxygen-deficient (V_O) and Eu-doped materials. From photon energies 5.2 eV to 7.2 eV, the refractive index increases with small fluctuations for the parent material, oxygen-deficient, and Eu-doped materials. The fluctuations occur due to various inner band transitions. Above 7.2 eV, the refractive index decreases for all materials. One can see that the graph of the oxygen-deficient refractive index is constant with small fluctuations, but in the visible region, a sudden decrease is observed in their values from 2 eV to 2.1 eV.

The optical activities are considered suitable for *n*(*ω*) values ranging from 2.0 to 4.0,^51^ and the materials under investigation fall within this range. The observation of refractive index values exceeding 1 strongly supports the semiconducting nature of these materials. Additionally, the refractive index provides insight into bond strength, with values above unity indicating stronger covalent bonding compared to ionic interactions. The presence of covalent bonds, whether static or dynamic, in these materials is attributed to photon deceleration and an increase in electron density.^52^

It can be expressed by means of the following relation
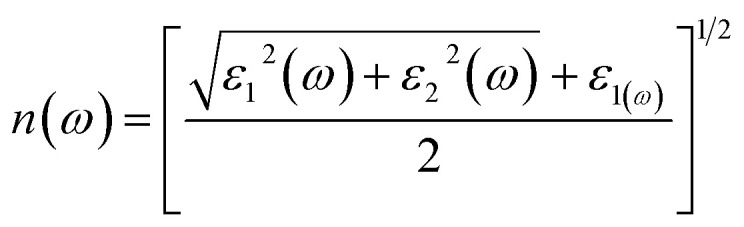
Equation describes the refractive index *n*(*ω*) of a material at a specific frequency *ω*, taking into account both the real and imaginary components of its permittivity. Here, *ε*_1_(*ω*) is the real part of the permittivity, related to the material's ability to store electric energy, while *ε*_2_(*ω*) is the imaginary part, corresponding to energy absorption. The equation combines these two components to compute the refractive index, which determines how light propagates through the material. This formulation is useful for understanding how materials interact with electromagnetic waves in optics and photonics.

## 4 Conclusion

We have reported the theoretical investigation of the structural and electronic properties of Eu-doped BaWO_4_ material and the creation of oxygen vacancies in the parent compound BaWO_4_, using the Full potential linearized augmented plane wave (FPLAPW) method, within the framework of Density Functional Theory (DFT). The elastic properties of BaWO_4_, including its elastic constants, bulk modulus, shear modulus, and Poisson's ratio, are significantly influenced by the introduction of oxygen vacancies and Eu doping. These modifications result in a predictable reduction in stiffness and rigidity but enhance the material's optoelectronic functionality. The calculations are made within the Generalized Gradient Approximation (GGA) + Optimized Effective Hubbard Parameter (*U*) (GGA + *U*) approach, employed in the WIEN2K software. Adding the Hubbard parameter *U* with a value of 7 eV provides a better description of the system with a strong correlation of d electronic states and f-electrons. To examine the electronic structure of Eu-doped BaWO_4_ material and the creation of oxygen vacancy (V_O) in the parent compound BaWO_4_, we have studied their band structures. The direct band gap is calculated for oxygen-deficient material, and the indirect band gap for Eu-doped BaWO_4_, with band gap values of 0.8 eV and 2.8 eV, respectively. We have computed the total, elemental, and partial densities of states (PDOS), and band structures for the optimization of the structure of Eu-doped BaWO_4_, which are discussed in detail. In partial density of states (PDOS), the strong influence of W: Ba: 4d, O, and Eu were noted as the foremost contributors in the hybridization process, thus enhancing the electronic and optical properties. The band structures and density of states reveal the semiconductor nature of the material. The structural properties, including bulk modulus, lattice constant (*a*), and cohesive energy, have been measured. For oxygen-deficient material, the least optical loss is seen in the visible region when the first maximum absorption peak is observed at 2 eV. In this region, the reflectivity is minimum, confirming that oxygen vacancy is beneficial for optoelectronic devices. Maximum reflectivity is observed in the ultraviolet region, but it is insufficient for use as a shielding material against ultraviolet radiation. The calculated results reveal that BaWO_4_: V_O is more suitable for optoelectronic devices due to its direct band gap semiconductor nature and smaller band gap values. The results calculated in our study are consistent with the literature.

## Data availability

All the data supporting the findings of this study are included in the article.

## Author contributions

All authors contributed equally in the article in conceptualization, investigation, analysis, writing original draft, review and editing.

## Conflicts of interest

The authors declare no competing interests.
