# Correction: Unveiling the enhanced structural, elastic, mechanical, and optoelectronic properties of BaWO_4_*via* oxygen vacancies and europium doping: a DFT + *U* insight into tailored energy applications

**DOI:** 10.1039/d5ra90081f

**Published:** 2025-07-08

**Authors:** Shah Hussain, Rajwali Khan, Sikander Azam, Qaiser Rafiq, Mehmoona Nisar, Wilayat Khan, Yasir Saeed, Mohammed A. Amin

**Affiliations:** a Department of Physics, Abbottabad University of Science and Technology Abbottabad Pakistan yasir.saeed@kaust.edu.sa; b National Water and Energy Center, United Arab Emirates University Al Ain 15551 United Arab Emirates; c Department of Physics, Riphah International University Islamabad Pakistan sikander.physicst@gmail.com; d Department of Physics, Bacha Khan University Charsada Pakistan; e Department of Chemistry, College of Science, Taif University P.O. Box 11099 Taif 21944 Saudi Arabia

## Abstract

Correction for ‘Unveiling the enhanced structural, elastic, mechanical, and optoelectronic properties of BaWO_4_*via* oxygen vacancies and europium doping: a DFT + *U* insight into tailored energy applications’ by Shah Hussain *et al.*, *RSC Adv.*, 2025, **15**, 18681–18696, https://doi.org/10.1039/D5RA01743B.

The authors regret that the name of one of the authors (Rajwali Khan) was shown incorrectly in the original article. The corrected author list is as shown above.

The Royal Society of Chemistry apologises for these errors and any consequent inconvenience to authors and readers.

